# A frameshift mutation of *TMPRSS3* in a Chinese family with non-syndromic hearing loss

**DOI:** 10.3389/fped.2022.1032659

**Published:** 2022-12-09

**Authors:** Jingwen Liang, Zhuoheng Yu, Zhangxing Wang, Jianxia Chen, Yihuan Liu, Zhaoqing Yin, Ruihuan Xu

**Affiliations:** ^1^ Clinical Laboratory, Longgang Central Hospital of Shenzhen, Shenzhen, China; ^2^ Division of Neonatology, Longhua People's Hospital, Shenzhen, China; ^3^ Clinical Laboratory, Shenzhen Mental Health Center, Shenzhen, China; ^4^ Division of Pediatrics, The People's Hospital of Dehong Autonomous Prefecture, Dehong Hospital of Kunming Medical University, Mangshi, Yunnan, China; ^5^ Department of Clinical Laboratory, The Second Affiliated Hospital, School of Medicine, The Chinese University of Hong Kong, Shenzhen, P. R. China & Longgang District People's Hospital of Shenzhen, Shenzhen, China

**Keywords:** TMPRSS3, homozygous, non-syndromic hearing loss, whole-exome sequencing, mutation

## Abstract

**Background:**

Deafness is the most common sensory defect in humans worldwide. Approximately 50% of cases are attributed to genetic factors, and about 70% are non-syndromic hearing loss (NSHL).

**Objectives:**

To identify clinically relevant gene variants associated with NSHL in a Chinese family using trio-based whole-exome sequencing (WES).

**Materials and methods:**

WES was performed on the 18-month-old female proband, and her parents. Gene variants specific to the family were identified by bioinformatics analysis and evaluated for their relevance to NSHL. We verified the novel variant in this family by the next-generation sequencing.In order to elucidate the frameshift mutation of *TMPRSS3* in a Chinese family, we used the Mass spectrometry to detect the gene from 1,010 healthy subjects.

**Results:**

We identified a novel homozygous deletion (c.51delA) in exon 2 of the type II transmembrane serine protease 3 gene *TMPRSS3*, which resulted in a frameshift mutation just before the protein transmembrane domain (*p*.Q17fs). The deletion was present in the proband and her father, but not in her mother and the healthy controls. We also found mutations with potential relevance to hearing loss in *DCAF17*, which encodes a protein of unknown function (c. T555A: *p*.H185Q), and *ZNF276*, which encodes zinc finger protein 276 (c.1350–2A > G).

**Conclusions and significance:**

We shown a novel frameshift mutation in *TMPRSS3* associated with autosomal recessive NSHL in a Han Chinese family.

## Introduction

Hearing loss is the most common sensory defect in humans worldwide. Genetic factors account for at least 50% of congenital hearing loss (1). About 80% of hereditary hearing impairment is due to autosomal recessive non-syndromic prelingual sensorineural hearing loss (2). Variants of the *TMRPSS3* gene have been associated with both familial and sporadic cases of autosomal recessive non-syndromic hearing loss (NSHL; DFNB8/10) (3). *TMPRSS3*, which encodes a transmembrane serine protease, is composed of 13 exons, with exon 2 containing the start codon (4). Many mutations in *TMPRSS3* have been reported to have potential roles in NSHL, including an 8-bp deletion, an insertion of multiple beta-satellite repeat units, and a frameshift mutation (4).

In our research, we studied the frameshift mutation of *TMPRSS3* that has thrown new light on the cause of the autosomal recessive NSHL in a Chinese patient.

## Materials and methods

The proband was an 18-month-old girl with NSHL. Her gestation period and birth weight (3.15 kg) were both normal, and she did not experience either asphyxia or hypoxia during delivery. Her postnatal development was normal, with head-raising at 3 months, sitting at 6 months, and walking at 14 months of age, with the exception of a small head circumference (44.5 cm) recorded at 18 months of age. General physical check-ups performed at standard times were normal. However, she was unable to speak and a hearing impairment was detected at 18 months of age. The child was diagnosed with profound sensorineural hearing loss at a local hospital*.* The parents have normal hearing and claim no family history of deafness or consanguinity.

To identify gene mutations that are candidates for the proband's deafness, we obtained peripheral blood samples (2 ml) from the proband, both parents, and 1,010 healthy subjects for DNA analysis. Genomic DNA was extracted using a Protease K DNA extraction kit (D3392), and the DNA was purified (OD260/280 = 1.8–2.0) and stored at −20°C until analysis.

**Figure 1:**
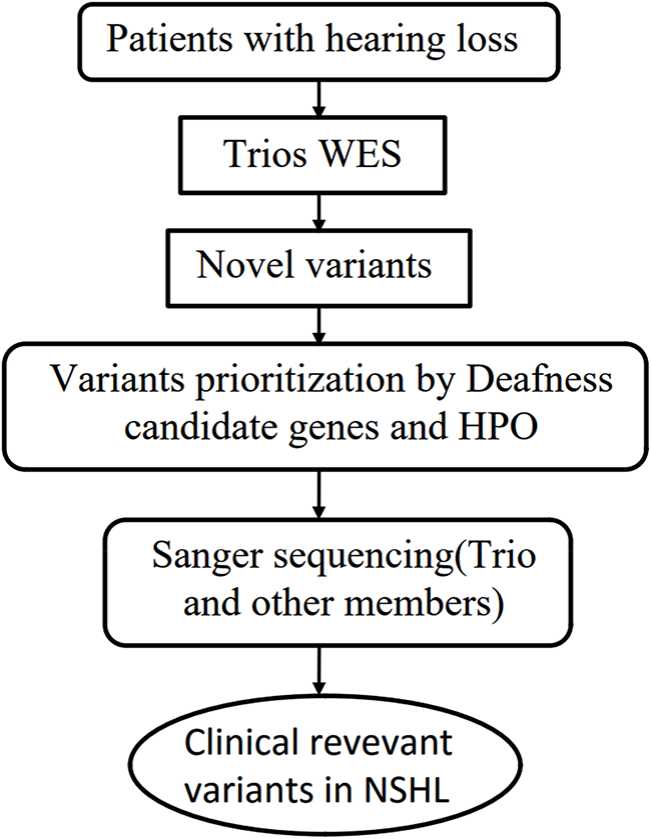
Flowchart to detect rare variants relevant to NSHL by WES. Abbrebiation: WES, Whole Exome Sequencing, HPO, Human Phenotype Ontology.

We performed whole-exome sequencing (WES) on the proband, her parents, and detected the gene locus in a large cohort of healthy controls by mass spectrometry.Variants identified in the proband were detected using trio-based bioinformatics analysis and evaluated for their potential association with NSHL by comparison with a PubMed database search. Candidate variants were then validated by sequencing of the proband and her family (Figure 1).

## Results

**Figure 2:**
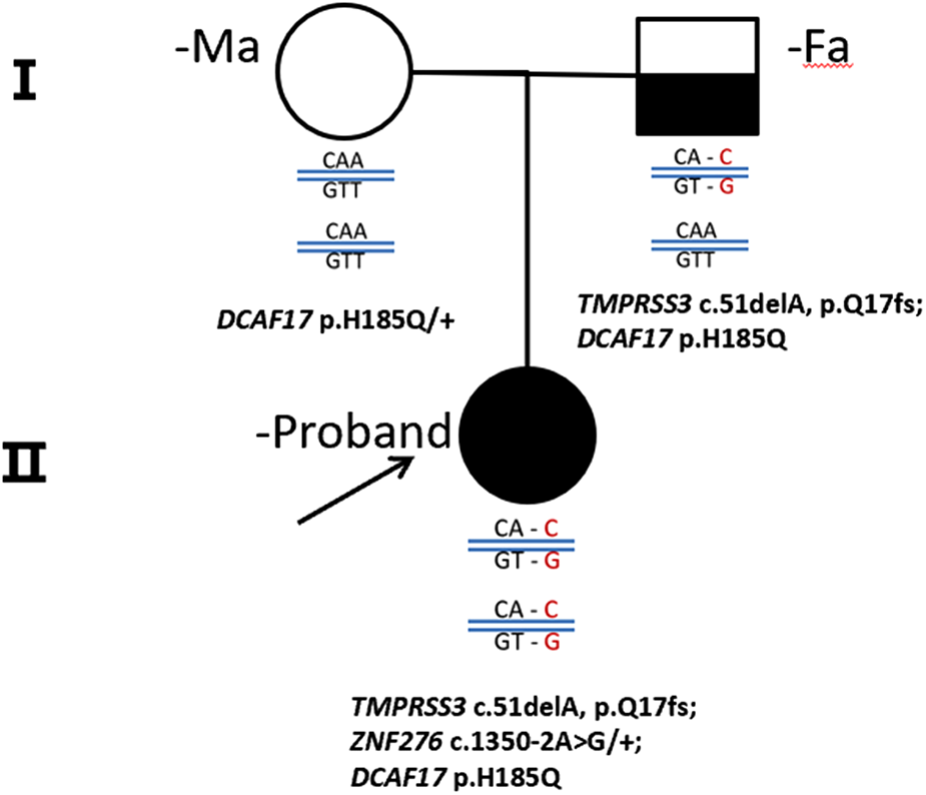
A pedigree drawings of the family with non-syndromic hearing imparemt. The proband's parent showed Normal hearing audiograms, while the affected gilrl, carried homozygous mutation, showed profound sensorineural hearing loss.

We conducted whole exome sequencing (WES) on the proband affected by NSHL and her corresponding parents.A novel candidate loci of *TMPRSS3* was identified in this case. The variants of *TMPRSS3* that were subsequently confirmed by next-generation sequencing on the Chinese Family. Family pedigree is shown in Figure 2. The proband (II-1) and her parents (I-1, I-2) carried *DCAF17*: c. T555 > A (*p*.H185Q). The proband (II-1) and her father, but not her mother and the healthy contrlos, carried *TMPRSS3*: c.51delA (*p*.Q17fs), showed profound sensorineural hearing loss. *TMPRSS3*: c.51delA (rs780609668) is a frameshift variant located in exon 2 and is known to be associated with autosomal recessive NSHL.

## Discussion

Mutations in a number of genes have been shown to cause severe non-syndromic deafness, including autosomal recessive NSHL (DFNB8/10), which is characterised by bilateral, severe to profound hearing loss. Such mutations have been identified in Palestinian, Tunisian, Korean, Indian, Spanish, and Greek populations (4, 8, 9, 16).

**Figure 3:**
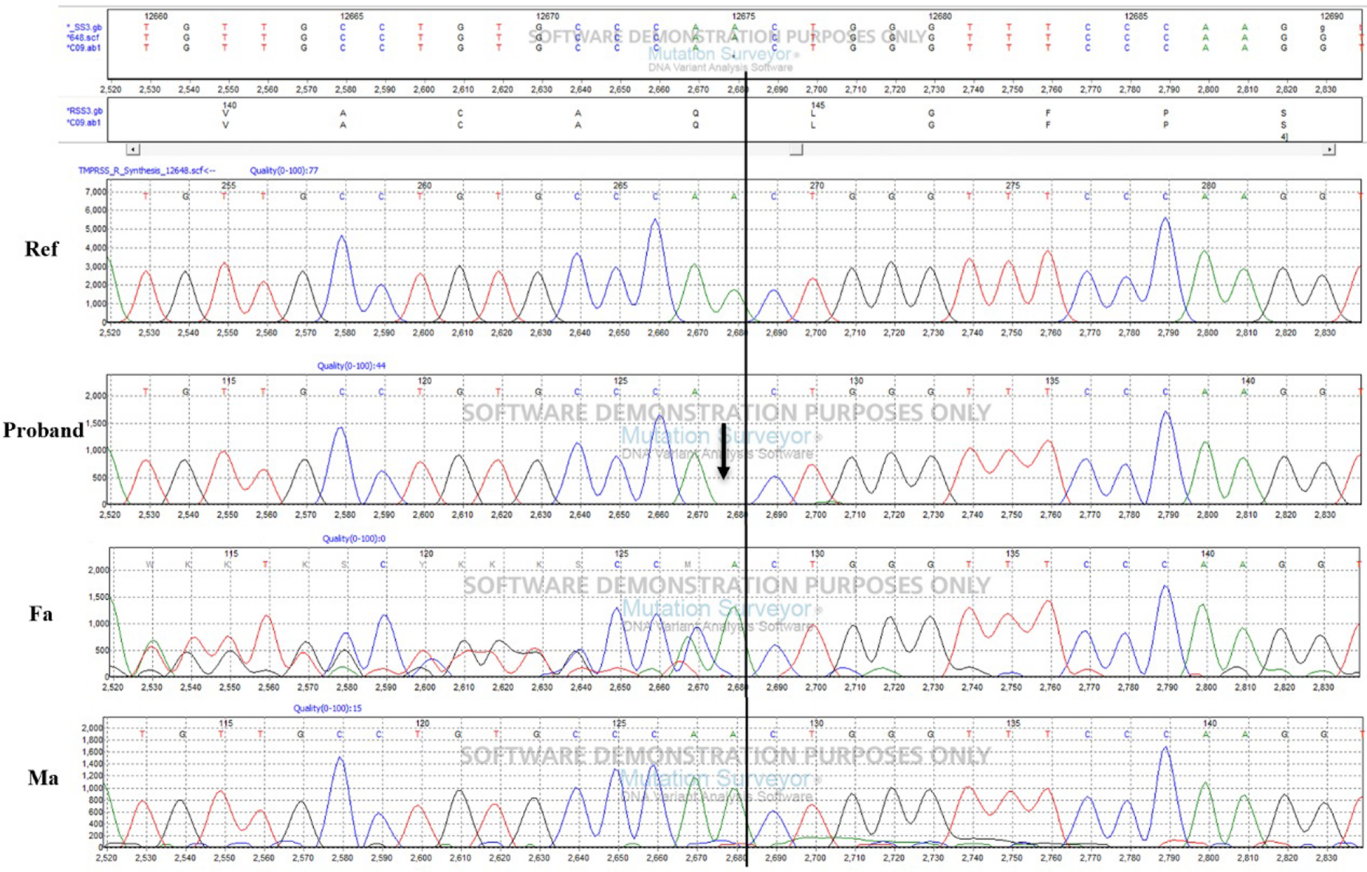
Validation of the frameshift mutation in *TMPRSS3* by sanger sequencing.

Our study found a frameshift mutation in *TMPRSS3* that causes autosomal recessive NSHL in a Chinese patient. The exon 2 deletion identified, c.51del, leads to a frameshift of glutamate at position 17 (*p*.Q17fs) in the protein. Confirmation of the putative disease-causing variant and analysis of *TMPRSS3* in all members of the Chinese family were performed by Sanger sequencing. The proband's father had a heterozygous *TMPRSS3* deletion, which leads to double tracing as shown in the left to the black line on Figure 3. Figure 1 shown that the proband had the homozygous deletion. The explanation would be a novel TMPRSS3 *p*.Q17fs mutation that occurred in her mother. But the proband's mother did not carry this frameshift *TMPRSS3 p*.Q17fs mutation. Maybe the proband's mother had a deletion that is spanning over the TMPRSS3 *p*.Q17fs exon (or several exons, which leads to no PCR amplification), which made the proband looked like to have the homozygous deletion. Not only that, the mutation of *TMPRSS3* was undetectable among 1,010 healthy subjects by Mass-spectrometric technique. These results indicate that the proband's deafness is likely to be caused by the homozygous *TMPRSS3*: c.51del mutation. The first mutation in a transmembrane protease associated with hearing loss was reported by Scott et al. in 2,001 (4). Since then, 24 mutations in *TMPRSS3* have been identified as potentially pathogenic for inheritable deafness (Table 1). Among these, 22 are missense mutations, one is a nonsense mutation, and one is a deletion. The *TMPRSS3 p*.Q17fs in our study would be very rare that frameshift mutation occurs in both alleles. Our discovery therefore adds another example of NSHL caused by a deletion mutation.

**Table 1:** Overview of the part of deafness-associated mutations in *TMPRSS3*.

Ethnicity	Amino Acid Change	Functional Domain	Type of Variant
China (this study)	Q17fs	Just before TM (11)	del
British (5)	Ala138Glu	SRCR	mis
Dutch (5)	Ala426Thr	TM	mis
Pakistan (6), Spanish (10)	Thr70fs	Truncation after TM	del
Tunisian (6)	Pro404Leu	Serine protease	mis
Korean (7), German (18)	Ala306Thr	TM	mis
Italian (8)	Thr340Arg	Serine protease	mis
Italian (8)	Pro431Ser	Serine protease	mis
Turkish (8)	Arg216Leu	TM	mis
Turkish (8)	Gln398X	Serine protease	mis
Tunisian (9)	Tpr251Cys	Serine protease	mis
Pakistani (16)	Cys407Arg	Serine protease	mis
Pakistani (16)	Cys194Phe	SRCR	mis
China Taiwan (17)	Arg80His	Serine protease	mis
China Taiwan (17)	Leu184Ser	Serine protease	mis
China Taiwan (17)	Ala418Val	LDLRA	mis
Greek (18)	Asp103Gly	LDLRA	mis
German (18)	Asp216Cys	Just before Serine protease	mis
Pakistani (19)	Cys425Arg	Serine protease	mis
Pakistani (19)	Glu104Lys	LDLRA	mis
Pakistani (19)	Glu104Stop	LDLRA	non
Pakistani (19)	Arg256Val	Serine protease	mis
Pakistani (19)	Arg109Trp	LDLRA	mis
UK Caucasian (20)	Ala90Thr	LDLRA	mis
Korean (21)	Thr248Met	Serine protease	mis

TM, transmembrane, SRCR, scavenger receptor cysteine-rich protein, LDLRA, low density lipoprotein receptor class A domains, del, deletion, mis, missense, Non, nonsynonymous.

We identified several other candidate deafness genes in the proband (Table 2). WES analyses showed that both parents were homozygous for *DCAF17*: c.T555 > A variant, and the proband was a carrier of the mutation. Mutations in *DCAF17* are associated with Woodhouse–Sakati syndrome, a rare disorder characterised by alopecia, hypogonadotropic hypogonadism, sensorineural hearing loss, diabetes mellitus, and extrapyramidal movement (12). Another study suggested that *DCAF17* may play as yet unexplored roles in tissue development and maintenance in adults (12). It is reported recently that *DCAF17* have played an important role in gametes growth and development (13). The development of embryonic chromosomes abnormalities is directly related to the quality of gametes.We identified a homozygous missense (c. T555>A: *p*.H185Q) in exon 6 of *DCAF17* in all members of the family (Table 2). *DCAF17*: c.555T > A has an allele frequency of 3% and is classified as a benign variant in the ClinVar database. Although, this variant does not associate with hearing loss. But, whether it was the homozygous missense matation in *DCAF17* that caused embryonic dysplasia resulting in deafness remained to be further studied.

**Table 2:** Candidate genes identified in the proband with NSHL.

Gene Symbol	Transcript	Exon	Nucleotide Change	Amino Acid Change	Position	Mode
*TMPRSS3*	NM_032404	Eexon2	c.51delA	*p*.Q17fs	Chr21-43,808,526	Hom
*DCAF17*	NM_001164821	Eexon6	c.T555 > A	*p*.H185Q	Chr2-172,309,651	Hom
*ZNF276*	NM_152287	–	c.1350-2A > G	–	Chr16-89,804,382	Het

In contrast, the affected girl was the only carrier of the mutation detected in *ZNF276* (c.1350–2A > G). The protein encoded by this gene is a 614-amino acid protein containing five C2H2-type zinc fingers and one zinc finger-associated domain. *ZNF276* mutations have been identified in breast cancer (14) and may also be involved in the progression of Fanconi anaemia (15).

In conclusion, we report a identification of a frameshift mutation in the *TMPRSS3* gene (c.51delA) resulting in a frameshift mutation in the protein (*p*.Q17fs) in the Han Chinese population. Our results thus provide a new example of autosomal recessive NSHL caused by a *TMPRSS3* mutation.

## Data Availability

The datasets presented in this study can be found in online repositories. The names of the repository/repositories and accession number(s) can be found in the article/Supplementary Material.
